# Sex‐dependent least toxic timing of irinotecan combined with chronomodulated chemotherapy for metastatic colorectal cancer: Randomized multicenter EORTC 05011 trial

**DOI:** 10.1002/cam4.3056

**Published:** 2020-04-22

**Authors:** Pasquale F. Innominato, Annabelle Ballesta, Qi Huang, Christian Focan, Philippe Chollet, Abdoulaye Karaboué, Sylvie Giacchetti, Mohamed Bouchahda, René Adam, Carlo Garufi, Francis A. Lévi

**Affiliations:** ^1^ North Wales Cancer Centre Betsi Cadwaladr University Health Board Bangor United Kingdom; ^2^ Division of Biomedical Sciences Cancer Chronotherapy Team Cancer Research Centre Warwick Medical School Coventry United Kingdom; ^3^ UMRS 935, "Cancer Chronotherapy and Postoperative Liver Functions" French National Institute for Health and Medical Research (INSERM) and Paris‐Sud University Villejuif France; ^4^ Department of Statistics University of Warwick Coventry United Kingdom; ^5^ Department of Oncology Clinique Saint‐Joseph CHC‐Liège Hospital Group Liège Belgium; ^6^ Clinical and Translational Research Division Jean Perrin Comprehensive Cancer Centre Clermont‐Ferrand France; ^7^ Medical Oncology Unit GHI Le Raincy‐Montfermeil Montfermeil France; ^8^ Department of Oncology Saint Louis Hospital Public Hospitals of Paris (AP‐HP) Paris France; ^9^ Mousseau Clinics Evry France; ^10^ Chronotherapy Unit Department of Medical Oncology Paul Brousse Hospital Public Hospitals of Paris (AP‐HP) Villejuif France; ^11^ Hepatobiliary Centre Paul Brousse Hospital AP‐HP Villejuif France; ^12^ Division of Medical Oncology San Camillo Forlanini Hospital Roma Italy

**Keywords:** chronotherapy, circadian, colorectal cancer, gender, irinotecan, toxicity

## Abstract

The least toxic time (LTT) of irinotecan varied by up to 8 hours according to sex and genetic background in mice. The translational relevance was investigated within a randomized trial dataset, where no LTT stood out significantly in the whole population.

130 male and 63 female eligible patients with metastatic colorectal cancer were randomized to receive chronomodulated Irinotecan with peak delivery rate at 1 of 6 clock hours staggered by 4 hours on day 1, then fixed‐time chronomodulated Fluorouracil‐Leucovorin‐Oxaliplatin for 4 days, q3 weeks. The sex‐specific circadian characteristics of grade (G) 3‐4 toxicities were mapped with cosinor and time*sex interactions confirmed with Fisher's exact test.

Baseline characteristics of male or female patients were similar in the six treatment groups. Main grade 3‐4 toxicities over six courses were diarrhea (males vs females, 39.2%; vs 46.0%), neutropenia (15.6% vs 15.0%), fatigue (11.5% vs 15.9%), and anorexia (10.0% vs 7.8%). They were reduced following irinotecan peak delivery in the morning for males, but in the afternoon for females, with statistically significant rhythms (*P* < .05 from cosinor) and sex*timing interactions (Fisher's exact test, diarrhea, *P* = .023; neutropenia, *P* = .015; fatigue, *P* = .062; anorexia, *P* = .032). Irinotecan timing was most critical for females, with grades 3‐4 ranging from 55.2% of the patients (morning) to 29.4% (afternoon) for diarrhea, and from 25.9% (morning) to 0% (afternoon) for neutropenia.

The study results support irinotecan administration in the morning for males and in the afternoon for females, in order to minimize adverse events without impairing efficacy.

## Graphical abstract

In this randomized international trial, the hematologic and clinical toxicities of irinotecan were least following dosing in the morning for men and in the afternoon for women with colorectal cancer. Sex‐specific least toxic times of anticancer drugs need further identification in prospective clinical trials.

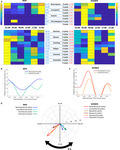

## 1 INTRODUCTION

Up to fivefold differences in systemic or tissue‐specific toxicities have been shown as a function of timing of administration for 50 anticancer drugs in experimental models.^1^, ^2^, ^3^, ^4^ The rhythms in anticancer drugs pharmacology are controlled by molecular clocks, whose suppression impairs the therapeutic benefits due to optimal treatment timing.^5^, ^6^ For instance, the lethal toxicity of irinotecan, a topoisomerase I inhibitor, was twice as large in male mice dosed at night, that is, near the middle of their nocturnal activity span, as compared to drug dosing in the second half of their resting span during daytime.^7^ Subsequent studies, however, revealed that the least toxic time of administration of irinotecan occurred about 6 hours later in female mice as compared to male mice.^8^ Additionally, large sex‐dependent differences in irinotecan chronopharmacology and chronotoxicity were observed and predicted by the reciprocal transcription dynamics of core clock genes Bmal1 and Rev‐Erbα.^8^, ^9^ Moreover, clinical evidence for sex‐related risk of toxicity on irinotecan and other drugs has been reported.^10^, ^11^, ^12^, ^13^ The triplet combination of irinotecan (I), 5‐Fluorouracil‐Leucovorin (FL), and Oxaliplatin (O), including its chronomodulation (chrono), has fostered forefront medico‐surgical strategies and enhanced survival and cures in patients with metastatic colorectal or pancreatic cancers.^14^, ^15^, ^16^, ^17^, ^18^ Nonetheless, the toxicity rates of such triplet combinations are nearly twice as large as those from doublets^15^ especially in female patients.^19^

Moreover, the efficacy of a fixed chronoFLO schedule prolonged overall survival as compared to constant rate FLO or FOLFOX in male but not in female patients.^20^ Neutropenia was halved on chronoFLO, yet being worse in female as compared to male patients on either schedule.^21^ In a time‐finding study comparing eight time‐lagged chronoFLO protocols in metastatic colorectal cancer patients, toxicity was nearly twice as large in female as compared to males. Moreover, optimal chemotherapy timing occurred 6 hours later in women as compared to men.^22^ The combination of irinotecan and chronoFLO proved to be active and safe in patients with colorectal cancer.^23^, ^24^ The current international, randomized, controlled, multi‐arm, time‐finding study aimed at the determination of the least toxic time of irinotecan, based on an expected rate of severe toxicities in 40% to 80% of the patients and no sex differences in time‐related toxic events.^25^ None of these hypotheses was validated, and their adequacy was subsequently questioned by preclinical and clinical reports.^8^, ^9^, ^10^, ^11^, ^12^, ^13^ Here, we show that irinotecan tolerability was largely and significantly improved following its delivery in the morning for males and in the afternoon for females with metastatic colorectal cancer.

## 2 PATIENTS AND METHODS

### 2.1 Patients

Adult patients with histologically proven, measurable and unresectable advanced colorectal cancer were eligible, if having a Performance Status (PS) of 0‐2, according to the classification of the World Health Organisation. They could have received up to one previous chemotherapy protocol (Supplementary text). All enrolled patients provided signed informed consent.

### 2.2 Study design

The protocol was approved by the three National Ethical Review Boards, and abided by the Helsinki Declaration's recommendations.^26^ The main objective was to assess the role of the time of irinotecan delivery, combined with fixed‐time chronoFLO.^24^ It was hypothesized that irinotecan timing would account for a 15% difference in the rate of patients with at least one toxicity‐related dose reduction or treatment delay over the initial three courses of chemotherapy irrespective of sex or prior treatment. It was calculated that the random allocation of 30 patients in each of the six corresponding irinotecan timing groups would enable the estimation of the least toxic time of irinotecan with 95% Confidence Interval of <6 hours, using a logistic regression model.^27^

**Figure 1:**
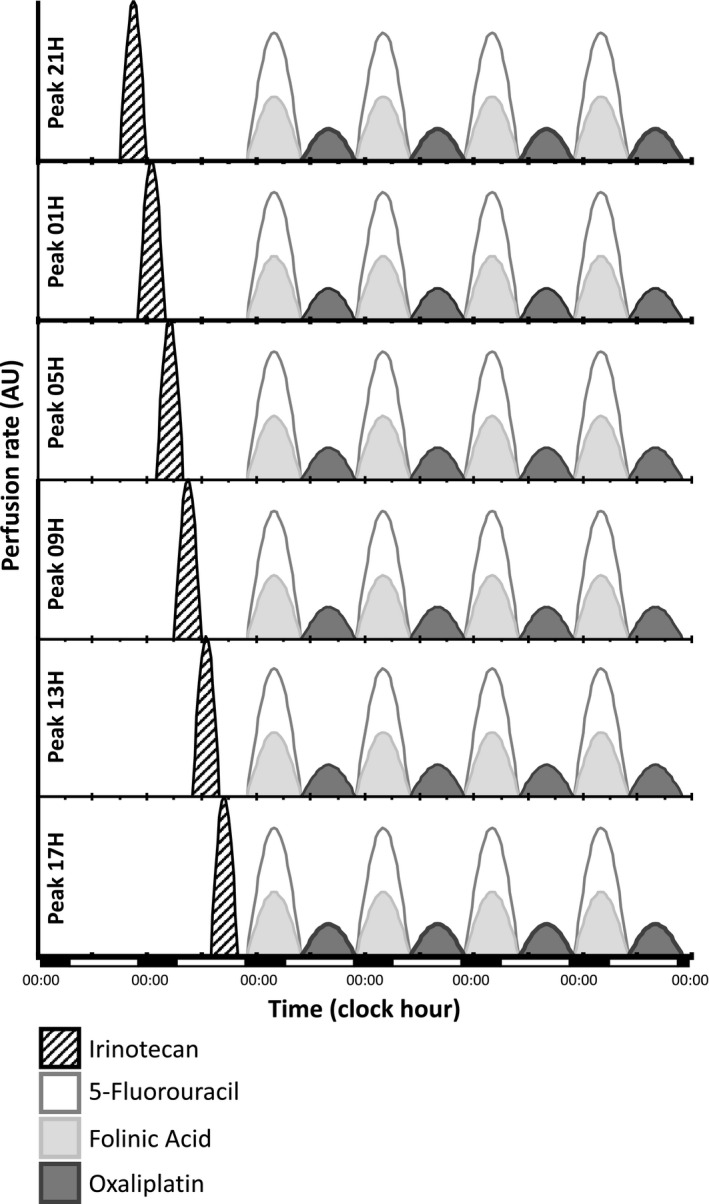
Graphical representations of the six treatment schemes compared. The triplet combinations consisted of day 1 chronomodulated irinotecan (180 mg/m^2^) over 6 h with 1 of 6 possible clock hours for infusion onsets and peak delivery rates (black, striped), followed by the same fixed time chronoFLO schedule. The latter involved days 2‐5 chronomodulated 5‐fluorouracil‐leucovorin from 22:15 to 9:45 (white and light gray, respectively), alternating with Oxaliplatin from 10:15 to 21:45 (dark gray) over 4 d. Cumulated doses per course were 2.8 g/m^2^ for 5‐Fluorouracil, 1.2g/m^2^ for leucovorin, and 80 mg/m^2^ for oxaliplatin. Courses were repeated every 21 d, that is, after a 16 d' interval. The abscissa indicates actual clock hours and days

Patients were randomized to receive irinotecan (180 mg/m^2^) as a 6‐hours chronomodulated infusion, with peak delivery times scheduled at 01:00, 05:00, 09:00, 13:00, 17:00, or 21:00 (Figure 1). All treatments were administered to nonhospitalized patients using a programmable‐in time, ambulatory infusion pump (Melodie, Aguettant).

Randomization was performed at the EORTC Data Center, with a minimization technique used for treatment allocation, stratifying by institution, PS (0‐1 vs 2), and line of treatment (1st vs 2nd), but not sex. Treatment allocation was indicated to the center following patient registration.

Hematological toxicity was assessed with weekly blood cell counts. Clinical and biochemical toxicities were assessed and graded according to the NCIC CTAE v2 criteria every 3 weeks. Treatment responses were assessed every third course based on computed thoraco‐abdomino‐pelvic tomography scan, and other relevant imaging techniques. Protocol treatment was to be continued until disease progression or confirmed complete response, treatment intolerance, or patient refusal (see also Supplementary Text about Trial Methods).

### 2.3 Endpoints

The primary endpoint of the trial was the proportion of patients presenting any clinical or hematological toxicity graded according to the Common Toxicity Criteria for Adverse Events v2 and requiring dose reduction or treatment delay, over the initial three courses, as a function of irinotecan timing. Objective responses (ORs) were assessed with the RECIST v1.1 criteria.^28^ The current report evaluates these outcomes separately in men and women, based on recent preclinical results and data from earlier clinical studies.^20^

### 2.4 Statistical analysis

Here, we first considered the proportions of male or female patients who experienced grade 3‐4 or grade 2‐4 toxicities within a given irinotecan timing schedule over the initial three or six cycles. We graphically displayed the sex‐specific relative changes for each main toxicity endpoint using a heat map, in order to visualize any consistent 24‐hour pattern across different toxicity endpoints for each sex. We then analyzed these data with cosinor.^29^ This nonlinear technique involved the fitting of a cosine curve with a period of 24 and/or 12 hours to the distribution of the toxicity data according to irinotecan peak delivery timing.^29^ The program computed the mesor (rhythm‐adjusted mean), the amplitude (half of the extent of the predictable change of the modeled curve) and the acrophase and bathyphase (respective times of peak and through of the modeled curve) with their respective 95% confidence limits. For each toxicity endpoint, F‐tests were used to determine the best‐fit model between (a) a flat line (ie, no rhythm) (b) a cosine curve with a period of 24‐hours with a 12‐hour harmonic, (c) a purely 24‐hour cosine curve, (d) a purely 12‐hour cosine curve. Statistical significance level was set to *P* < .05. This was programmed using Matlab (The Mathworks, Inc).

Clinically relevant implications were visually highlighted further through pooling toxicity or efficacy data corresponding to irinotecan dosing in the morning (peak delivery time at 05:00 or 09:00; midpoint, 07:00), in the afternoon (peak delivery time at 13:00 or 17:00; midpoint, 15:00) or at night (peak delivery time at 21:00 or 01:00; midpoint, 23:00), separately for male and female patients. Sex*irinotecan timing interactions were further analyzed using Chi‐Square and Fisher's exact test. All toxicity analyses were performed on the treated and evaluated patients. Efficacy was assessed in the eligible patient population using SPSS v24 (IBM Inc). Statistical significance level was set at *P* < .05.

## 3 RESULTS

### 3.1 Patient characteristics

**Figure 2:**
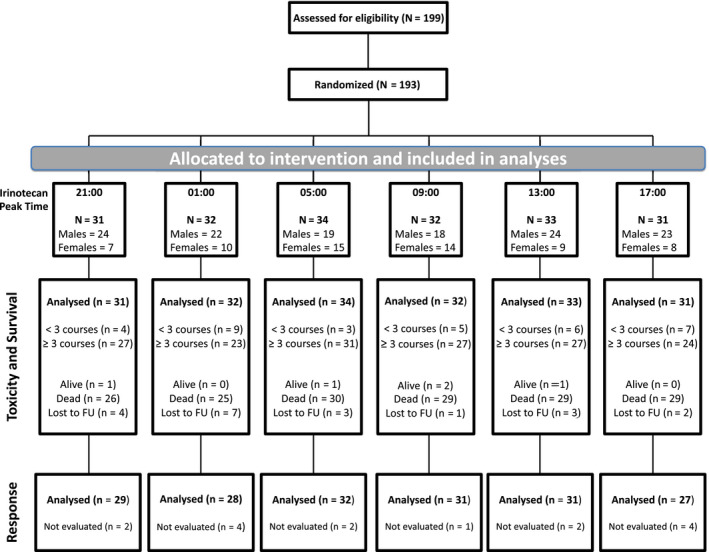
CONSORT diagram. Six patients were ineligible because of concomitant cancer or inappropriate cancer staging

From February 2002 to August 2006, 199 patients were enrolled at 18 institutions in Belgium, France or Italy, and randomized to one of the six treatment groups as planned (Figure 2). The eligible study population involved 193 patients (97%). Two eligible patients received irinotecan according to a protocol modality that differed from the one assigned by randomization and were reallocated to the real treatment timing group. ChronoIFLO was administered as first‐line treatment to 149 patients (77.2%) and as second line to 44 patients (22.8%). The main clinical and demographic features of the eligible patient population were similar in the six treatment groups. Overall, study participants included 130 males and 63 females, with a PS of 0 for 142 patients (73.5%) and a median age of 61 years (range: 29‐80). There were 18 to 24 males and 7 to 15 females in each treatment modality, with similar clinical characteristics among each treatment modality (Table 1). Likewise, the occurrence of comorbidities, the incidence of metastatic stage at diagnosis and the proportion of patients having received adjuvant chemotherapy were comparable in both men and women among the six treatment groups (data not shown). Nonsignificant imbalances were seen for the percentages of patients with two or more metastatic sites.

**Table 1:** Main clinical features of the eligible study population, separately according to sex and to randomized treatment modality as defined by timing of irinotecan peak delivery rate

Males	Modality							
	All N = 130	01:00 n = 22	05:00 n = 19	09:00 n = 18	13:00 n = 24	17:00 n = 23	21:00 n = 24	*P*‐value
Age, (y)								
Median (range)	62 (33‐80)	64 (51‐76)	62 (46‐80)	60 (45‐70)	62.5 (33‐77)	62 (50‐79)	61.5 (38‐71)	.492
PS (WHO)								
0	97 (74.6%)	14 (63.6%)	16 (84.2%)	14 (77.8%)	15 (62.5%)	20 (87.0%)	18 (75.0%)	
1	29 (22.3%)	7 (31.8%)	3 (15.8%)	3 (16.7%)	8 (33.3%)	2 (8.7%)	6 (25.0%)	
2	4 (3.1%)	1 (4.5%)	0	1 (5.6%)	1 (4.2%)	1 (4.3%	0	.441
Site of primary tumor								
Colon	104 (80.0%)	17 (77.3%)	17 (89.5%)	16 (88.9%)	19 (79.2%)	16 (69.6%)	19 (79.2%)	
Rectum	26 (20.0%)	5 (22.7%)	2 (10.5%)	2 (11.1%)	5 (20.8%)	7 (30.4%)	5 (20.8%)	.691
Prior chemotherapy								
No	97 (74.6%)	18 (81.8%)	13 (68.4%)	14 (77.8%)	17 (70.8%)	18 (78.3%)	17 (70.8%)	.906
N of sites involved								
1	66 (50.8%)	8 (36.4%)	15 (78.9%)	8 (44.4%)	12 (50.0%)	11 (47.8%)	12 (50.0%)	
2	43 (33.1%)	7 (31.8%)	2 (10.5%)	9 (50.0%)	7 (29.2%)	9 (39.1%)	9 (37.5%)	
3 or more	21 (16.2%)	7 (31.8%)	2 (10.5%)	1 (5.6%)	5 (20.8%)	3 (13.0%)	3 (12.5%)	.168
Sites involved								
Liver only	49 (37.7%)	8 (36.4%)	13 (68.4%)	4 (22.2%)	7 (29.2%)	7 (30.4%)	10 (41.7%)	
Liver + other	59 (45.4%)	12 (54.5%)	3 (15.8%)	10 (55.6%)	11 (45.8%)	12 (52.2%)	11 (45.8%)	
Other only	22 (16.9%)	2 (9.1%)	3 (15.8%)	4 (22.2%)	6 (25.0%)	4 (17.4%)	3 (12.5%)	.173

Females	Modality							
	All N = 63	01:00 n = 10	05:00 n = 15	09:00 n = 14	13:00 n = 9	17:00 n = 8	21:00 n = 7	*P*‐value
Age, years								
Median (range)	58 (29‐77)	58.5 (35‐77)	59 (31‐70)	56 (49‐76)	58 (34‐70)	51.5 (42‐74)	54 (29‐70)	.741
PS (WHO)								
0	45 (71.4%)	9 (90.0%)	9 (60.0%)	9 (64.3%)	7 (77.8%)	6 (75.0%)	5 (71.4%)	
1	15 (23.8%)	1 (10.0%)	5 (33.3%)	5 (35.7%)	1 (11.1%)	1 (12.5%)	2 (28.6%)	
2	3 (4.8%)	0	1 (6.7%)	0	1 (11.1%)	1 (12.5%)	0	.666
Site of primary tumor								
Colon	49 (77.8%)	8 (80.0%)	11 (73.3%)	12 (85.7%)	6 (66.7%)	6 (75.0%)	6 (85.7%)	
Rectum	14 (22.2%)	2 (20.0%)	4 (26.7%)	2 (14.3%)	3 (33.3%)	2 (25.0%)	1 (14.3%)	.642
Prior chemotherapy								
No	52 (82.5%)	8 (80.0%)	13 (86.7%)	11 (78.6%)	7 (77.8%)	7 (87.5%)	6 (85.7%)	.988
N of sites involved								
1	27 (42.9%)	3 (30.0%)	3 (20.0%)	6 (42.9%)	6 (66.7%)	4 (50.0%)	5 (71.4%)	
2	20 (31.7%)	4 (40.0%)	5 (33.3%)	6 (42.9%)	0	3 (37.5%)	2 (28.6%)	
3 or more	16 (25.4%)	3 (30.0%)	7 (46.7%)	2 (14.3%)	3 (33.3%)	1 (12.5%)	0	.117
Sites involved								
Liver only	19 (30.2%)	3 (30.0%)	1 (6.7%)	3 (21.4%)	5 (55.6%)	4 (50.0%)	3 (42.9%)	
Liver + other	29 (46.0%)	6 (60.0%)	10 (66.7%)	6 (42.9%)	2 (22.2%)	3 (37.5%)	2 (28.6%)	
Other only	15 (23.8%)	1 (10.0%)	4 (26.7%)	5 (35.7%)	2 (22.2%)	1 (12.5%)	2 (28.6%)	.245

### 3.2 Overall treatment toxicities and dose intensities

Four patients died during the initial 2 months (2.1%), including two toxic deaths (1%). A 76 y.o male patient died with grade 5 diarrhea after the first course involving irinotecan peak delivery at 01:00; a 50 y.o. male patient died with a grade 5 gastro‐intestinal fistula and circulatory collapse after two courses where irinotecan peak delivery was scheduled at 17:00. The Independent Data Monitoring Committee confirmed the safety of the protocol, with an observed toxicity not exceeding the expected range after inclusion of 100 patients.

A total of 1,138 courses were administered, with a median of six per patient (range: 1 to 18). The analysis of the drug delivery report outputs revealed the occurrence of pump dysfunction resulting in underdosing for 2.8% of the courses. The main reasons for protocol discontinuation were toxicity (37.8%), disease progression (23.8%), complete response after surgery of metastases (10.9%), or patient refusal (8.3%). Three or more protocol courses were administered to 82.3% of the male patients, and 82.5% of the female patients, without any obvious difference according to treatment group (Table S1).

Over the initial six treatment courses, grades 3‐4 diarrhea was reported for 51/130 men (39.2%) and 29/63 women (46%). Grades 3‐4 neutropenia was encountered in 20/128 men (15.6%) and 9/60 women (15%) (NS). Grade 3 fatigue was experienced by 15/130 men (10%) and 10/63 women (15.9%). Grade 3 anorexia occurred in 13/130 men (10%) and 5/63 women (7.9%), while grade 3‐4 anemia was found for 6/60 women (10%), as compared to 1/127 men (0.8%).

Median dose intensities (mg/m^2^/week) were 53 for irinotecan, 24 for oxaliplatin, and 853 and 199 for 5‐fluorouracil and leucovorin over the whole treatment span, and without any significant difference according to sex and prior chemotherapy (not shown). These figures corresponded to actual relative dose intensities > 70% of those planned for 93.4% to 94.9% of the patients according to the drug considered.

The percentages of patients withdrawn for toxicity according to irinotecan timing had a similar range in males (from 25% to 47.8%) and in females (from 28.6% to 50%), This rate was highest at 17:00 for male patients, as compared to 09:00 for female patients (Table S1).

### 3.3 Relevance of irinotecan timing according to sex

**Figure 3:**
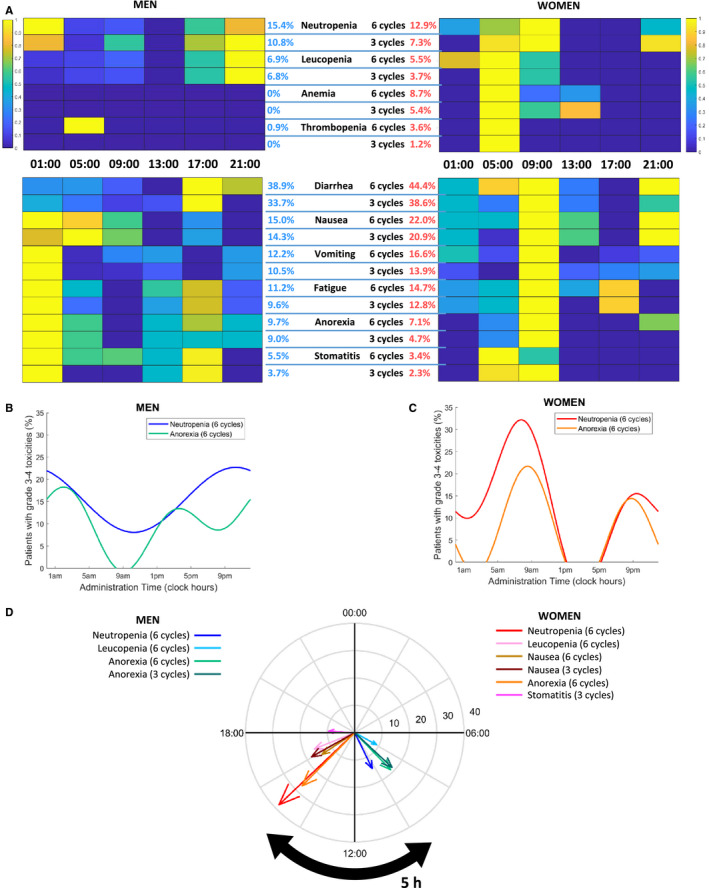
Main chronoIFLO toxicities according to irinotecan timing and sex. A, Heat maps of percentages of male or female patients having developed grade 3‐4 hematological or clinical toxicities over three or six cycles. B, Best fit cosine curves for percentages of men with grade 3‐4 neutropenia or anorexia over six cycles with respect to irinotecan timing. C, Best fit cosine curves for percentages of women with grade 3‐4 neutropenia or anorexia over six cycles with respect to irinotecan timing. D, Polar plots highlighting the sex‐specific clock hours associated with minimum incidence of toxicities. For each toxicity, the arrow's length and angle represent amplitude and clock time of minimum value of the best‐fit cosine, respectively. Only toxicities with significant cosinor tests are displayed (*P* < .05)

As shown in heatmap graphs (Figure 3A), grade 3‐4 leukopenia and neutropenia were worst in the male patients receiving irinotecan with peak delivery time at 21:00 and least at 13:00 over the initial three or six courses. In contrast for the female patients, the most toxic times corresponded to 05:00 or 09:00, while treatment was least hematotoxic following irinotecan delivery with peak rate at 17:00. Clinical tolerability was best in males but worst in females following irinotecan peak delivery rate at 09:00. Irinotecan peak delivery rate at 13:00 or 17:00 resulted in best clinical tolerability in women.

Cosinor analysis revealed statistically significant circadian rhythms in the proportions of patients with grade 3 or 4 toxicities according to irinotecan timing for four endpoints in men, and for six of them in women (Figure 3B,C, Tables S2 and S3). Twenty‐four hour or (24 + 12 hours) rhythms were found for neutropenia, leukopenia, and anorexia over six cycles both in men and in women, yet with distinct waveforms and circadian parameters. Thus, the double amplitudes were 9.1% in men vs 15.9% in women for leukopenia, 14.6% in men vs 38.3% in women for neutropenia, and 18.9% in men vs 27.3% in women for anorexia (six courses). The least toxic timing of irinotecan peak delivery rate was located in the morning hours in men, being 07:55 for leukopenia, 9:04 for anorexia, and 10:16 for neutropenia (six cycles). In women, the corresponding optimal times were located in the afternoon, that is, 16:32, 14:59, and 15:06 (Figure 3D, Table S3). Female patients further displayed a (24 + 12‐hours) rhythm in stomatitis and a 12‐h rhythm in nausea. Optimal administration times were very consistent across all toxicity types for women, with times of minimum of simulated toxicity curves ranging from 14:48 to 16:03.

### 3.4 Antitumor efficacy

Response rates, progression‐free survival or overall survival did not differ according to sex or irinotecan peak delivery rate timing (Table S4), with overall respective figures of 56.7% [95% CL, 49.4 to 64.0], 8.4 months [7.5‐9.3] and 19.2 months [15.4‐22.9].

Cosinor analysis did not show any significant rhythm for best overall response rates, response rates at first evaluation, progression‐free survival or overall survival either in men (*P* = .41, .24, .32 or .29, respectively) or in women (*P* = .47, .16, .29, or .15, respectively).

### 3.5 Practical implications for optimization of irinotecan timing

**Figure 4:**
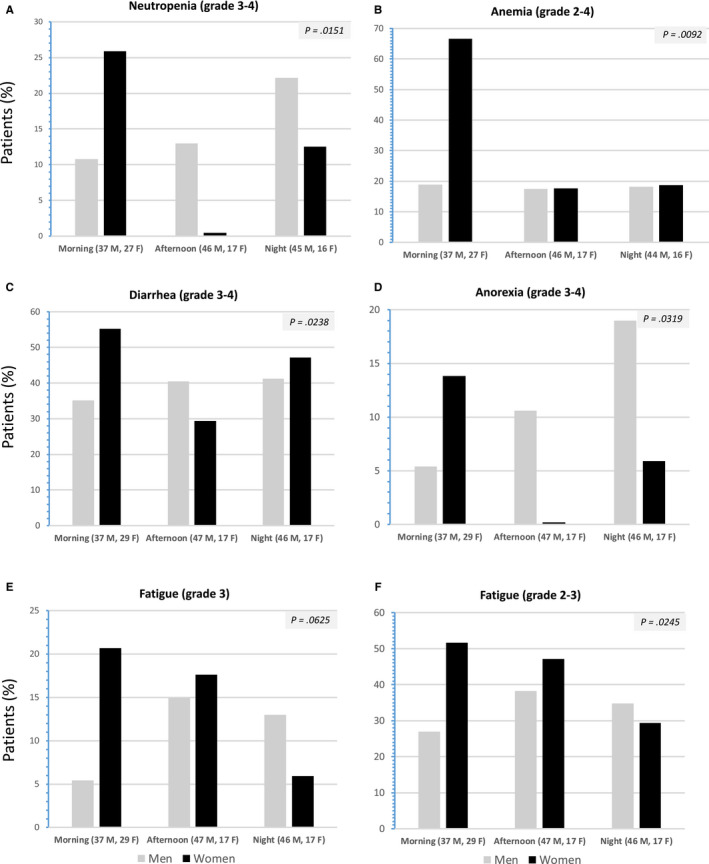
Histograms depicting the incidence of the main severe toxicities over the initial six courses of chronoIFLO, in male or female patients as a function of peak delivery rate of irinotecan occurring in the morning (05:00 or 09:00), in the afternoon (13:00‐17:00), or at night (21:00‐01:00). A, Neutropenia (grades 3‐4); B, Anemia (grades 2‐4); C, Diarrhea (grades 3‐4); D, Anorexia; (grades 3‐4); E and F, Fatigue (grade 3, and grades 2‐3, respectively). The displayed p‐values from Fisher's exact test correspond to the statistical significance of sex*timing interactions from Fisher's exact test for each endpoint

Statistically significant interactions between sex and timing of irinotecan peak delivery rate (morning vs afternoon vs night) were validated for the incidences of grades 2‐4 and grades 3‐4 toxicities using Fisher's exact test (Figure 4). Overall, tolerability was best following peak delivery of irinotecan in the morning for males and in the afternoon for females.

**Figure 5:**
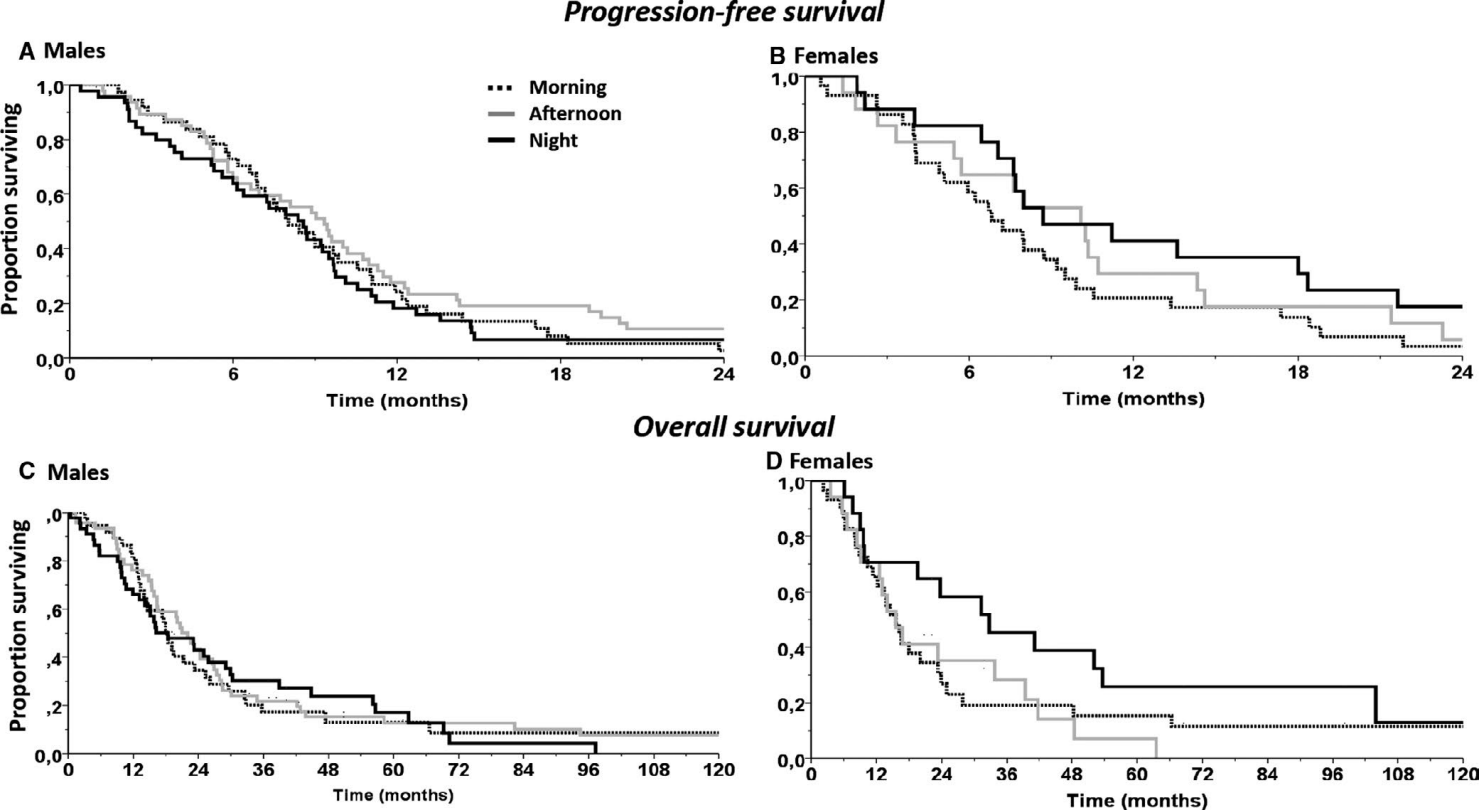
Efficacy of chronoIFLO according to irinotecan timing in the morning, in the afternoon or at night in each sex. Progression‐free survival curves in (A) male patients and (B) female patients. Overall survival curves according to irinotecan timing in (C) male patients and (D) female patients (see Table S3 for statistical comparisons)

In contrast, no significant difference was found regarding progression‐free survival or overall survival according to morning vs afternoon vs night timing of irinotecan both in male and in female patients (Figure 5).

## 4 DISCUSSION

The current study has revealed a sex‐specific least toxic time of irinotecan administration with regard to objectively assessed neutropenia and other hematologic and clinical endpoints, in an international randomized trial, despite the least toxic time of administration of irinotecan could not be identified in the whole population. The incidence of severe neutropenia varied nearly threefold according to irinotecan timing in each sex. The least neutropenic dosing time of irinotecan was located in the morning for men and in the afternoon for women. The other toxicity endpoints followed 24‐hour variations that matched those of neutropenia, thus impacting on clinical tolerability, and treatment compliance. The magnitude of the time related differences in toxicities support the integration of optimal treatment timing specifications into the core of precision cancer medicine.^1^, ^30^

Optimal irinotecan timing could theoretically be personalized even more accurately through systems medicine.^1^, ^31^ Thus, coupled experimental chronopharmacology data of irinotecan and their mathematical modeling have identified the main molecular clock determinants of chronotolerance.^6^, ^31^ The dynamic assessment of circadian function before, during, and after chemotherapy administration, can further streamline such personalized chronotherapy through circadian biomarkers tele‐monitoring.^32^, ^33^, ^34^ The safety of chronoIFLO delivery at home was recently highlighted, through the remote and continuous joint monitoring of circadian biomarkers and patient‐reported outcome measures with a dedicated eHealth platform.^34^

The current trial has revealed that the patient's sex was an important factor that could alter optimal circadian timing of chemotherapy, thus confirming earlier findings with chronoFLO. Indeed, statistically significant differences according to sex were demonstrated for progression‐free survival and overall survival in each of three international randomized Phase III trials and their meta‐analysis in 842 patients with metastatic colorectal cancer receiving fixed‐time chronoFLO. Men on this treatment protocol displayed both significantly less toxicity and significantly better survival, as compared to women on the same schedule, independently from other prognostic factors.^20^, ^21^ Suggestive evidence for an about 6‐hour difference in optimal timing of chronoFLO between men and women was further provided in a time‐finding clinical trial involving 114 patients. The daily timing of the chronoFLO schedule, that was here combined with irinotecan, was on the average, the least toxic one for men, but not for women.^22^ The sex differences in the circadian patterns in chemotherapy tolerability were further supported by the demonstration of (a) sexual dimorphism in molecular circadian clock,^35^ (b) the molecular clock control of irinotecan cellular pharmacology,^6^ and (c) the sex dependency of irinotecan optimal timing and pharmacology in mice, resulting in a 4‐ to 6‐hour delay in females as compared to males.^8^, ^9^, ^36^, ^37^ It was unlikely that sexual hormones played a major role in the sex‐dependent differences in outcomes, given the fact that the vast majority of women were postmenopausal, with a median age of 58 years. Conversely, sex‐specific differences in both the endogenous circadian period and the phase angle of entrainment of circadian clocks have been evidenced in controlled laboratory studies in humans.^38^, ^39^ Alongside sex‐related circadian dissimilarities, other biological differences between men and women, including immune system activity, body composition, pharmacology of anticancer drugs, or the microbiome have been hypothesized as modulators of sex‐dependent differences in treatment toxicity.^40^

A limitation in this study involves the post‐hoc analysis of prospective data, with no a priori power calculation or stratification based on sex subgroups. Notwithstanding, no significant differences in patients' characteristics were observed. Its strength lies on the validation of the hypothesis that was generated after the study had been completed, due to the subsequent discovery of sex‐specific optimal irinotecan timing in mice.^8^, ^9^, ^36^, ^37^

In summary, this time‐finding trial has identified sex‐specific times for optimizing irinotecan tolerability, that is, in the morning hours for men, and in the afternoon hours for women. Such timing specifications deserve prospective validation. ChronoIFLO delivery could readily be personalized thanks to mHealth platforms in the home setting, which allow for remote patients monitoring and real time determinations of individual circadian phase and patient condition.^33^, ^34^, ^41^, ^42^, ^43^ The framework of systems pharmacology and systems medicine offers advanced mathematical means for optimizing chronotherapy according to molecular circadian clocks in individual patients.^1^

## CONFLICT OF INTEREST

All authors declare no conflict of interest.

## AUTHOR CONTRIBUTION

CG and FL designed, conducted, and coordinated the clinical trial. AB, QH, and AK performed the mathematical and statistical analyses. PI, CF, PC, SG, MB, RA, CG, and FL included patients and gathered the majority of the data. FL, PI, and AB wrote the manuscript. All authors discussed, read, and approved the manuscript.

## Supporting information

Table S1

Table S2

Table S3

Table S4

Supplementary Material

## Data Availability

The data that support the findings of this study are available from the corresponding author upon reasonable request. This work is under Creative Common CC‐BY.
