# Supplementary material for: Sex‐dependent least toxic timing of irinotecan combined with chronomodulated chemotherapy for metastatic colorectal cancer: Randomized multicenter EORTC 05011 trial
Source: Cancer Med. 2020 Apr 22;9(12):4148–59. doi: 10.1002/cam4.3056 (PMC7300418; doi:10.1002/cam4.3056)
Supplement: Supplementary file 1 — Table S1 [file CAM4-9-4148-s001.docx]

**TABLE S1 – Main reported causes of protocol withdrawal according to sex and peak delivery time of irinotecan (modality).**

| Cause | Sex | Modalities | | | | | | | |
| --- | --- | --- | --- | --- | --- | --- | --- | --- | --- |
|  |  | All | 01 :00 | 05 :00 | 09 :00 | 13 :00 | 17 :00 | 21 :00 | P from  Fisher-Exact |
|  |  | N events/  N of pts (%) | N events/  N of pts (%) | N events/  N of pts (%) | N events/  N of pts (%) | N events/  N of pts (%) | N events/  N of pts (%) | N events/  N of pts (%) |  |
| Toxicity | Males | 47/130 (36.2%) | 9/22 (40.9%) | 7/19 (36.8%) | 6/18 (33.3%) | 6/24 (25.0%) | 11/23 (47.8%) | 8/24 (33.3%) | 0.697 |
|  | Females | 26/63 (41.3%) | 4/10 (40.0%) | 7/15 (46.7%) | 7/14 (50.0%) | 3/9 (33.3%) | 3/8 (37.5%) | 2/7 (28.6%) | 0.941 |
|  | All | 73/193 (37.8%) | 13/32 (40.6%) | 14/34 (41.2%) | 13/32 (40.6%) | 9/33 (27.3%) | 14/31 (45.2%) | 10/31 (32.3%) | 0.691 |
| Progression | Males | 31/130 (23.8%) | 4/22 (18.2%) | 6/19 (31.6%) | 4/18 (22.2%) | 11/24 (45.8%) | 1/23 (4.3%) | 5/24 (20.8%) | 0.026 |
|  | Females | 15/63 (23.8%) | 4/10 (40.0%) | 5/15 (33.3%) | 2/14 (14.3%) | 2/9 (22.2%) | 2/8 (25.0%) | 0 | 0.433 |
|  | All | 46/193 (23.8%) | 8/32 (25.0%) | 11/34 (32.4%) | 6/32 (18.8%) | 13/33 (39.4%) | 3/31 (9.7%) | 5/31 (16.1%) | 0.058 |
| Complete response | Males | 5/130 (3.8%) | 1/22 (4.5%) | 2/19 (10.5%) | 1/18 (5.6%) | 0 | 0 | 1/24 (4.2%) | 0.450 |
|  | Females | 1/63 (1.6%) | 0 | 0 | 0 | 0 | 0 | 1/7 (14.3%) | 0.111 |
|  | All | 6/193 (3.1%) | 1/32 (3.1%) | 2/34 (5.9%) | 1/32 (3.1%) | 0 | 0 | 2/31 (6.5%) | 0.618 |
| Patient refusal | Males | 11/130 (8.5%) | 1/22 (4.5%) | 0 | 3/18 (16.7%) | 1/24 (4.2%) | 5/23 (21.7%) | 1/24 (4.2%) | 0.086 |
|  | Females | 5/63 (7.9%) | 0 | 0 | 1/14 (7.1%) | 1/9 (11.1%) | 1/8 (12.5%) | 2/7 (28.6%) | 0.157 |
|  | All | 16/193 (8.3%) | 1/32 (3.1%) | 0 | 4/32 (12.5%) | 2/33 (6.1%) | 6/31 (19.4%) | 3/31 (9.7%) | 0.054 |
| Metastases surgery | Males | 13/130 (10.0%) | 0 | 1/19 (5.3%) | 2/18 (11.1%) | 2/24 (8.3%) | 3/23 (10.0%) | 5/24 (20.8%) | 0.263 |
|  | Females | 8/63 (12.7%) | 1/10 (10.0%) | 1/15 (6.7%) | 2/14 (14.3%) | 1/9 (11.1%) | 1/8 (12.5%) | 2/7 (28.6%) | 0.837 |
|  | All | 21/193 (10.9%) | 1/32 (3.1%) | 2/34 (5.9%) | 4/32 (12.5%) | 3/33 (9.1%) | 4/31 (12.9%) | 7/31 (22.6%) | 0.215 |
| Lost to follow-up | Males | 1/130 (0.8%) | 1/22 (4.5%) | 0 | 0 | 0 | 0 | 0 | 0.454 |
|  | Females | 0 | 0 | 0 | 0 | 0 | 0 | 0 | - |
|  | All | 1/193 (0.5%) | 1/32 (3.1%) | 0 | 0 | 0 | 0 | 0 | 0.653 |
| Other causes | Males | 21/130 (16.2%) | 5/22 (22.7%) | 3/19 (15.8%) | 2/18 (11.1%) | 4/24 (16.7%) | 3/23 (13.0%) | 4/24 (16.7%) | 0.951 |
|  | Females | 8/63 (12.7%) | 1/10 (10.0%) | 2/15 (13.3%) | 2/14 (14.3%) | 2/9 (22.2%) | 1/8 (12.5%) | 0 | 0.939 |
|  | All | 29/193 (15.0%) | 6/32 (18.8%) | 5/34 (14.7%) | 4/32 (12.5%) | 6/33 (18.2%) | 4/31 (12.9%) | 4/31 (12.9%) | 0.966 |
