# Supplementary material for: Sex‐dependent least toxic timing of irinotecan combined with chronomodulated chemotherapy for metastatic colorectal cancer: Randomized multicenter EORTC 05011 trial
Source: Cancer Med. 2020 Apr 22;9(12):4148–59. doi: 10.1002/cam4.3056 (PMC7300418; doi:10.1002/cam4.3056)
Supplement: Supplementary file 2 — Table S2 [file CAM4-9-4148-s002.docx]

**Table S2: Results from cosinor analyses of main Grade 3-4 toxicities of irinotecan-based chemotherapy in the whole population of eligible patients.**

Data are the rates of patients with Grade 3-4 hematologic or clinical toxicities over 3 or 6 courses according to irinotecan peak time of delivery. Periods of 24-h and/or 12 h were fit to the data, and the best fitting model was statistically tested in comparison to the lack of any rhythmic pattern. Cosinor parameters included the mesor and the rhythm parameters including the amplitude relative to mesor (with mesor being normalized as 1), and the acrophase or time of maximum in hours and decimal hours; for cosine fits with a 24-h or a 12 h period , and the time of maximum and minimum toxicities of the simulated curve, as well as its double amplitude. All parameters are given with their corresponding SD
