# Supplementary material for: Sex‐dependent least toxic timing of irinotecan combined with chronomodulated chemotherapy for metastatic colorectal cancer: Randomized multicenter EORTC 05011 trial
Source: Cancer Med. 2020 Apr 22;9(12):4148–59. doi: 10.1002/cam4.3056 (PMC7300418; doi:10.1002/cam4.3056)
Supplement: Supplementary file 3 — Table S3 [file CAM4-9-4148-s003.docx]

**Table S3:** **Results from cosinor analyses of main Grade 3-4 toxicities of irinotecan-based chemotherapy (a) in male patients and (b) in female patients.**

1. **Male patients**

1. **Female patients**
