# Supplementary material for: Sex‐dependent least toxic timing of irinotecan combined with chronomodulated chemotherapy for metastatic colorectal cancer: Randomized multicenter EORTC 05011 trial
Source: Cancer Med. 2020 Apr 22;9(12):4148–59. doi: 10.1002/cam4.3056 (PMC7300418; doi:10.1002/cam4.3056)
Supplement: Supplementary file 4 — Table S4 [file CAM4-9-4148-s004.docx]

**Table S4: Efficacy endpoints according to irinotecan peak delivery time in male (a) or female (b) patients**

1. **Males**

|  | **01AM** n=22 | **05AM** n=19 | **09AM** n=18 | **13PM** n=24 | **17PM** n=23 | **21PM** n=24 | **All** N=130 |
| --- | --- | --- | --- | --- | --- | --- | --- |
| **Best overall response** |  |  |  |  |  |  |  |
| CR | 1 (4.5%) | 2 (10.5%) | 1 (5.6%) | 0 | 1 (4.3%) | 1 (4.2%) | 6 (4.6%) |
| PR | 8 (36.4%) | 5 (26.3%) | 12 (66.7%) | 11 (45.8%) | 14 (60.9%) | 11 (45.8%) | 61 (46.9%) |
| SD | 6 (27.3%) | 10 (52.6%) | 3 (16.7%) | 7 (29.2%) | 4 (17.4%) | 7 (29.2%) | 37 (28.5%) |
| PD | 3 (13.6%) | 1 (5.3%) | 2 (11.1%) | 4 (16.7%) | 0 | 4 (16.7%) | 14 (10.8%) |
| Not assessed | 4 (18.2%) | 1 (5.3%) | 0 | 2 (8.3%) | 4 (17.4%) | 1 (4.2%) | 12 (9.2%) |
| **Objective response** |  |  |  |  |  |  |  |
| Yes | 9 (50.0%) | 7 (38.9%) | 13 (72.2%) | 11 (50.0%) | 15 (78.9%) | 12 (52.2%) | 67 (56.8%) |
| No | 9 (50.0%) | 11 (61.1%) | 5 (27.8%) | 11 (50.0%) | 4 (21.1%) | 11 (47.8%) | 51 (43.2%) |
| **PFS** |  |  |  |  |  |  |  |
| Median [95%CL] | 8.6  [5.2-11.9] | 7.9  [4.9-10.9] | 8.0  [6.3-9.7] | 6.1  [1.1-11.2] | 9.4  [7.9-11.0] | 8.4  [6.1-10.6] | 8.7  [7.5-9.8] |
| **OS** |  |  |  |  |  |  |  |
| Median [95%CL] | 16.2  [11.4-21.0] | 18.2  [15.9-20.5] | 14.4  [3.9-24.9] | 22.5  [18.6-26.5] | 20.1  [11.5-28.7] | 23.1  [7.1-39.1] | 19.4  [15.5-23.4] |

1. **Females**

|  | **01AM** n=10 | **05AM** n=15 | **09AM** n=14 | **13PM** n=9 | **17PM** n=8 | **21PM** n=7 | **All** N=63 |
| --- | --- | --- | --- | --- | --- | --- | --- |
| **Best overall response** |  |  |  |  |  |  |  |
| CR | 0 | 0 | 0 | 0 | 0 | 1 (14.3%) | 1 |
| PR | 5 (50.0%) | 10 (66.7%) | 7 (50.0%) | 4 (44.4%) | 3 (37.5%) | 4 (57.1%) | 33 (52.4%) |
| SD | 3 (30.0%) | 3 (20.0%) | 3 (21.4%) | 3 (33.3%) | 4 (50.0%) | 1 (14.3%) | 17 (27.0%) |
| PD | 2 (20.0%) | 1 (6.7%) | 3 (21.4%) | 2 (22.2%) | 1 (12.5%) | 0 | 9 (14.3%) |
| Not assessed | 0 | 1 (6.7%) | 1 (7.1%) | 0 | 0 | 1 (14.3%) | 3 (4.8%) |
| **Objective response** |  |  |  |  |  |  |  |
| Yes | 5 (50.0%) | 10 (71.4%) | 7 (53.8%) | 4 (44.4%) | 3 (37.5%) | 5 (83.3%) | 34 (56.7%) |
| No | 5 (50.0%) | 4 (28.6%) | 6 (46.2%) | 5 (55.6%) | 5 (62.5%) | 1 (16.7%) | 26 (43.3%) |
| **PFS** |  |  |  |  |  |  |  |
| Median [95%CL] | 7.1  [5.2-8.9] | 6.8  [4.1-9.5] | 6.7  [2.1-11.3] | 10.1  [3.8-16.3] | 7.6  [0.0-19.6] | 13.6  [7.4-19.8] | 8.0  [6.5-9.4] |
| **OS** |  |  |  |  |  |  |  |
| Median [95%CL] | 9.8  [0.0-39.7] | 16.2  [12.6-19.8] | 12.2  [4.5-20.0] | 13.9  [11.3-16.5] | 16.8  [0.0-36.5] | 41.1  [19.8-62.3] | 16.8  [11.5-22.1] |
