# Supplementary material for: Sex‐dependent least toxic timing of irinotecan combined with chronomodulated chemotherapy for metastatic colorectal cancer: Randomized multicenter EORTC 05011 trial
Source: Cancer Med. 2020 Apr 22;9(12):4148–59. doi: 10.1002/cam4.3056 (PMC7300418; doi:10.1002/cam4.3056)
Supplement: Supplementary file 5 — Supplementary Material [file CAM4-9-4148-s005.docx]

**Supplementary Text about Trial Methods**

**Inclusion-non-inclusion criteria**

Patients could not have received the same triplet as that given in this trial for metastatic or recurrent disease. Adjuvant chemotherapy was considered as 1^st^ line treatment if relapse or metastasis occurred within 6 months from completion. Patients were required to have adequate hepatic, renal and haematological functions, no baseline diarrhoea > grade 1 (NCIC CTCAE v2), and no prior toxicity related to irinotecan ≥ grade 3. Patients with uncontrolled medical conditions, or psycho-social issues representing a potential risk for study compliance and for patient’s safety were excluded.

**Supportive treatments and dose or interval modifications**

Prophylactic corticosteroids or hematologic growth factors were not allowed unless previously documented severe clinical or hematologic toxicities, respectively.

In case of any grade 3-4 clinical or haematological toxicity, doses were reduced by 30 mg/m^2^ for irinotecan, 200 mg/m^2^ for 5-fluorouracil, and 20 mg/m^2^ for oxaliplatin. A single dose reduction step, possibly involving the three drugs, was allowed. The occurrence of a subsequent episode of grade 3-4 toxicity, despite appropriate dose reduction, resulted in patient’s withdrawal from protocol for toxicity. Treatment was delayed till recovery to grade <2 clinical and/or haematological toxicities. In case of persistent toxic event (i.e., grade ≥2 neutropenia, thrombocytopenia, diarrhoea, stomatitis or fatigue) preventing treatment administration beyond 28 days from the 1st day of the previous course, patient was withdrawn from protocol for toxicity.

**Endpoints**

The toxicities that were considered for treatment dose or interval modifications included neutropenia, thrombocytopenia, diarrhoea, stomatitis or asthenia ≥ grade 2 at 3 weeks after the beginning of the previous course, or any grade 3 or 4 toxicity occurred at any time.

Secondary endpoints included objective response rate, worst toxicity over the initial 6 courses and over the whole treatment span, and progression-free and overall survival durations. These endpoints were evaluated as a function of treatment group in the whole study eligible population.

Time to event endpoints were computed from the day of randomisation till progression or death, whichever occurred first (PFS), or till death or last date known to be alive (OS). Last date with valid follow-up data was used to censor non-progressing or alive patients, respectively.

**Statistical design**

The initial protocol was based on the hypothesis that the timing of irinotecan administration would account for a 15% difference in the rate of patients with at least one toxicity-related dose reduction or treatment delay over the initial 3 courses of chemotherapy. This difference was deemed as clinically meaningful, as 40 to 80% of the patients receiving the FOLFIRINOX chemotherapy protocol reportedly interrupted treatment for severe toxicity (23,24). Assuming a sinusoidal profile of irinotecan chronotolerance pattern, with a maximum and a minimum occurring 12 hours apart, it was calculated that six chronomodulated delivery modalities, with maximum delivery rates staggered by 4-hours would adequately test this hypothesis through the random allocation of 30 patients in each of the six corresponding groups. Such numbers were required to estimate the least toxic time of irinotecan administration with 95% Confidence Interval (95%CI) of < 6 hours. It was considered that neither sex nor prior chemotherapy would influence irinotecan timing effects on tolerability endpoints. Under such assumption, the estimation was based on a logistic regression model, as detailed in a previous similar randomised study (28).

**Data base**

Database was updated and frozen on November 2^nd^, 2016. Survival curves were estimated using the Kaplan-Meier technique, and compared with a log-rank test.
